# Screening of Key Fungal Strains in the Fermentation Process of the Chinese Medicinal Preparation “Lianzhifan Solution” Based on Metabolic Profiling and High-Throughput Sequencing Technology

**DOI:** 10.3389/fmicb.2021.727968

**Published:** 2021-08-23

**Authors:** Jie Xie, Yang Ye, Ze Wu, Xun Gou, Tong Peng, Xuegang Yuan, Xiangdong Yang, Xiaoyu Zhang, Quekun Peng

**Affiliations:** ^1^College of Life Sciences, Sichuan Normal University, Chengdu, China; ^2^Keystonecare Technology (Chengdu) Co., Ltd., Chengdu, China; ^3^Department of Anorectal Surgery, The Sixth People’s Hospital of Chengdu, Chengdu, China; ^4^Department of Anorectal, Chengdu Anorectal Hospital, Chengdu, China; ^5^Department of Biotechnology, School of Bioscience and Technology, Chengdu Medical College, Chengdu, China

**Keywords:** traditional Chinese medicine fermentation, key strain screening, equivalence evaluation, chemical metabolic profiling, iridoids, high-throughput sequencing

## Abstract

“Lianzhifan solution” (LZF) is produced by the natural fermentation of coptis root and gardenia fruit, and it is a classic prescription for external use in anorectal department. During the fermentation process, the structural evolution of microbial communities led to significant changes in the chemical profile. In this study, we first analyzed the dynamic changes of chemical components as well as the composition and succession of microbial community during the whole fermentation process of LZF, and confirmed the changes of characteristics of nine compounds during the whole fermentation process by metabolic profile. Further analysis found that there was no significant change of alkaloids in all stages of fermentation of LZF, but there were significant changes of iridoids in the middle and late stage of fermentation by deglycosylation. Genipin gentiobioside and geniposide were converted to genipin by biotransformation, showing that deglycosylation was the main event occurring in the fermentation. The community composition and abundance of species in 10 and 19days LZF fermentation broth were analyzed with high-throughput sequencing technology, and 16 dominant bacterial genera and 15 dominant fungal genera involved in the fermentation process were identified. Correlation analysis revealed that *Penicillium expansum* and *Aspergillus niger* involved in the fermentation were the dominant genera closely related to the dynamic changes of the deglycosylation of the main chemical components, and *P. expansum* YY-46 and *A. niger* YY-9 strains were obtained by the further fractionation. Then the monoculture fermentation process was evaluated, whereby we found that the deglycoside conversion rate of iridoid glycosides was greatly improved and the fermentation cycle was shortened by 3–4 times. This finding combined with equivalence evaluation of chemical component and pharmacodynamics to confirm that *P. expansum* YY-46 and *A. niger* YY-9 strains were key strains for fermentation concoction. This study established an efficient and practical screening strategy “Microfauna communities-Chemical component-Pharmacodynamic” axis for key strain, to improve the production process and formulating good manufacturing practice (GMP) work, and it is also applicable to the whole fermentation drugs industry.

**Graphical Abstract:**
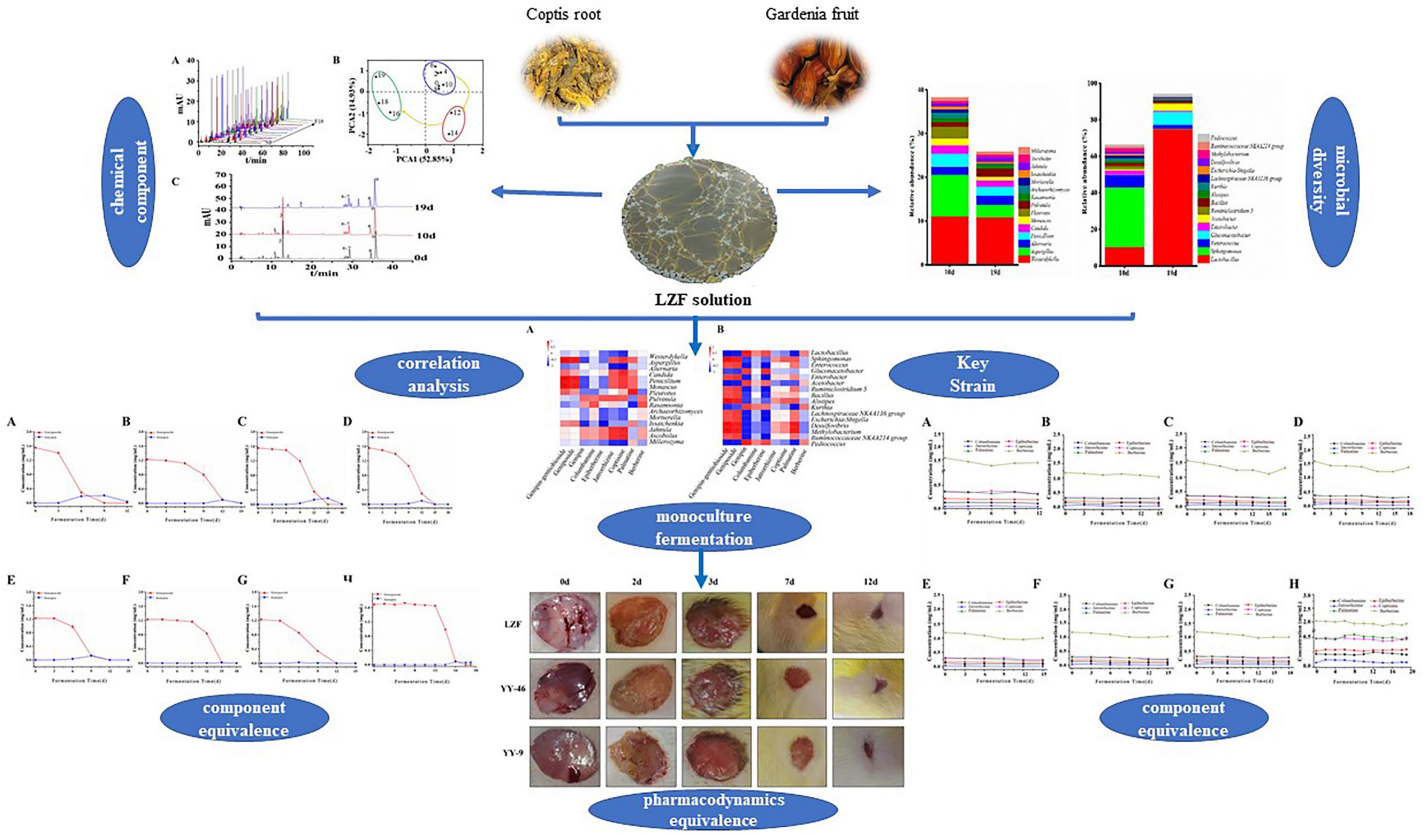
The figure highly summarizes the research content of this study and shows the screening process of key strains in LZF fermentation.

The figure highly summarizes the research content of this study and shows the screening process of key strains in LZF fermentation.

## Introduction

As early as more than a 1,000years ago, microbial fermentation has been applied to the concoction of traditional Chinese medicine (TCM) in China. Under certain environmental conditions (e.g., temperature, humidity, air, moisture, etc.) the chemical components of raw or processing drugs are transformed by microorganisms and this fermentation produces a variety of secondary metabolites (Li et al., 2020), new chemical components, or more active precursor compounds (Kim et al., 2014), and achieves new uses such as toxicity reduction and storage (Marič et al., 2019), potency enhancement (Zhang et al., 2017; Liu et al., 2018), and bioavailability improvement (Yin et al., 2017; Yim et al., 2018). Most of the traditional fermentation of TCM is natural fermentation processes using crude drugs in a natural environment with a mixture of microorganism, and it is so difficult to control the types and numbers of microorganisms involved in the fermentation environment that some “ineffective” or even “harmful” strains are involved. At the same time, this traditional fermentation method is not obviously transformation-oriented and does not control the changes of the main active ingredients of the TCM in the fermentation process. The control of the whole fermentation process generally relies only on the sensory and empirical judgment of workers. And the process lacks the support of mature, systematic and reliable scientific methods, resulting in the unstable quality of the whole traditional fermented concoction Chinese medicines, and hindering the application of large-scale production. Therefore, it is of great significance for industrial conversion of traditional processes to screen microorganisms involved in fermented concoction, to control active ingredients, to explore of the change pattern of ingredients and microorganisms and their correlation during the process of Chinese medicine concoction, and to clarify the mechanism of action of fermented concoction.

“Fermented Coptis chinensis solution,” also known as “Lianzhifan Solution” (LZF), is a classic formula for external use with a history of 3 centuries (Yuan et al., 2016). It has been used in Chengdu Anorectal Specialized Hospital for the prevention and treatment of perianal abscess, ulcerative colitis, and anorectal diseases in tens of thousands of patients. LZF consists of two herbs (coptis root and gardenia fruit) and traces of the mineral Chinese medicine alum. In fact, the coptis root is not really a root but the dried rhizome of *Coptis chinensis* Franch, a plant of the buttercup family, and the main active ingredients are alkaloids such as epiberberine, coptisine, and berberine (Dai et al., 2016); Gardenia fruit is the dried fruit of the *Gardenia jasminoides* Ellis and the main active ingredients are genoside, genipin-1-*β*-D-gentiobioside (Bergonzi et al., 2012). The production of LZF is an uncommon fermentation process, and the production process still strictly follows the production method determined by Mr. Jichuan Huang a 100years ago, in which the pharmacist decocted coptis root, gardenia fruit, and alum at 100°C according to the formula, and then the medicinal solution is filtered and left to ferment in a dark and humid place for 1–3weeks, and when the surface of the liquid shows a leopard print pattern (map spot pattern) of greenish-gray fungus and the reddish-brown color of the drug liquid, the experienced pharmacists confirm the ending point and then filter the drug solution before bottling it for clinical use. The production process is quite different from that of common topical tinctures, and is more similar to the fermentation process of soy sauce.

From the perspective of modern biotechnology, multi-strain liquid fermentation is the unique point of the production process of LZF, which is reflected in the fact that no other carbon and nitrogen sources are added. Our previous study revealed that the difference of chemical profile of different batches and different storage periods of the preparation may be related to the degree of transformation of its chemical composition by microorganisms (Ye et al., 2019). Therefore, the changes in the chemical composition of LZF are closely related to the action of microorganisms during the fermentation process. At the same time, the authors in tracking the production process of different production batches in year 2019 found that the surface microorganisms at the end of fermentation of each batch differed in morphology and color, etc. Sometimes green mold was dominant, sometimes tawny mold was dominant, but sometimes yeast film was dominant; and the fermentation time of different batches also differed: generally, 1–2weeks in spring and summer and 2–3weeks in autumn and winter. This difference suggests that the composition of microorganisms involved in the preparation process may vary from batch to batch, or the duration of microbial conversion may vary. The group used high-throughput sequencing technology to determine and analyze the diversity and richness of fungi during the fermentation and preparation of LZF, and found that the overall trend of the richness and diversity of fungi increased gradually with the increase of fermentation time (Yuan et al., 2018). In recent years, studies on the effect of fermentation on the microecological community structure of TCM have also been reported (Shimoyama et al., 2015; Huang et al., 2020; Qu et al., 2021). For example, some scholars have analyzed the microbial diversity of Shenqu at different fermentation times by PCR-DGGE (Liu et al., 2017). Some scholars have also used *Aspergillus* spp. to ferment cardiovascular drugs (as statins; Barrios-González et al., 2020; Al-Saman et al., 2021).

At present, LZF preparation still relies on pharmacist’s personal observation and experience to control the fermentation process, which had risks of quality control and could hardly meet the good manufacturing practices (GMP) requirements for modern drug production. Therefore, it is necessary to use modern biotechnology to deeply analyze the quality control index components and dominant bacterial groups of this preparation and to identify the key strains of fermented preparations. For this purpose, this study used quantitative metabolic profile mapping to analyze the dynamic changes of chemical components during the fermentation of LZF, combined with high-throughput sequencing technology to understand the dominant microorganisms in the fermentation process. Then this study screened the strains by step-by-step isolation, and further used chemical component and bioequivalence evaluation to identify the key functional strains. This will provide important and strong scientific support for improving the traditional empirical formulation model, optimizing the formulation process, enhancing product quality and stability, and establishing GMP work.

## Materials and Methods

### Materials and Reagents

Coptis root, the dried rhizome of *Coptis chinensis* Franch (batch #470330), gardenia fruit, the dried fruit of *G. jasminoides* Ellis (batch #470401), and Alunite (batch #151121) were purchased from Chengdu Kangmei Pharmaceutical Manufacturing Co., Ltd., and were identified by prof. Xiaoyu Zhang. Genipin-1-*β*-D-gentiobioside (Cas# 29307-60-6), Geniposide (Cas# 24512-63-8), Genipin (Cas# 6902-77-8), Columbamine (Cas# 3621-36-1), Epiberberine (Cas# 6873-09-2), Jatrorrhizine (Cas# 3621-38-3), Coptisine (Cas# 3486-66-6), Palmatine (Cas# 3486-67-7), and Berberine (Cas# 2086-83-1) were purchased from Chengdu Ruifensi Biotechnology Co., Ltd.; Chromatographic pure acetonitrile, methanol, hydrochloric acid, and phosphoric acid were purchased from Chengdu ShuoboYanchuang Science & Technology Co., Ltd.

EB (Sangon Biotech, E607322); Agarose (Sangon Biotech, A600234); PCR enzyme (KOD-401B: TOYOBO KOD-Plus-Neo DNA Polymerase); DNA marker (Takara, DL2000); TE Buffer; The primers of ITS2 region areITS3_KYO2 (5′-GATGAAGAACGYAGYRAA-3′) and ITS4 (5′-TCCTCCGCTTATTGATATGC-3′); The primers of 16S rDNA V4 region are 515F (5′-GTGYCAGCMGCCGCGGTAA-3′) and 806R (5′-GGACTACHVGGGTWTCTAAT-3′; Caporaso et al., 2011); DNA Isolation Kit (MO BIO PowerSoil DNA Isolation Kit); Gel Extraction Kit (Omega); Sequencing library kit (TruSeq DNA PCR-Free Sample Prep Kit); and On-line sequencing kit (Hiseq Rapid SBS Kit V2).

### Instruments and Equipments

High performance liquid chromatography (HPLC; Agilent, 1200); UPLC-PDA-ESI-MS (Waters, H-Class QDa); UV Spectrophotometer (Thermo Fisher, NanoDrop2000C BioMate3S); E-Gel Imager (Bio-Rad, VersaDoc 5000); Centrifuge (Eppendorf, 5424R); Electrophoresis System (Bio-Rad, Powerpac Basic1645050); Fluorescent quantizer (Invitrogen, Qubit 2.0); Gel extractor (OMEGA-Biotek, Firefly NIMBUS® 96); PCR instrument (ABI, Applied Biosystems GeneAmp 9700); Sequenator (Illumina, Hiseq 2500); and Biological Analyzer (Agilent, 2100).

### LZF Preparation and Sampling

The coptis root, gardenia fruit, and trace amount of the mineral Chinese medicine alum were decocted according to the formula. The filtrate was then collected in a fermentation barrel placed under the cover at room temperature for natural fermentation. Samples of the upper and lower layers of fermentation broth were taken at 0, 2, 4, 6, 8, 10, 12, 14, 16, 18, and 19days. Around 20ml liquid was taken with a sterile pipette from 5cm below the liquid surface at each of the five points, and these five samples were then mixed to form the 100ml upper fermentation broth. Then, 20ml liquid was taken with a sterile pipette from 10cm above the bottom of the barrel at each of the five points, and these five samples were then mixed to form the 100ml lower fermentation broth. Finally, the upper and lower fermentation broths were mixed at ratio of 1:1.

The chromatographic analysis samples were prepared as follows: 100μl of the mixed sample was added with 100μl of hydrochloric acid and 9,800μl of methanol, treated with ultrasonic for 5min, and then filtered with 0.22μm filter, and the chromatographic analysis sample was obtained. Finally, the chemical constituents of the solution were analyzed by HPLC and ultra performance liquid chromatography (UPLC)-MS. In addition, on 10 and 19days of fermentation, the fermentation mixture was taken for high-throughput sequencing.

### HPLC Analysis Chemical Composition Changes During the Fermentation Process of LZF

HPLC analysis was performed on an Agilent 1200 HPLC system (Agilent, United States). System control and data analysis were performed on the Chemstation Software program (version A.10.02). The separation was performed on an Eclipse column, Agilent C18 (4.6×250mm, 5μm). The binary gradient consisted of 0.1% phosphoric acid in distilled water (*v*/*v*; solvent A) and acetonitrile (solvent B), following the elution program: 0min (5% B), 8min (15% B), 15min (18% B), 20min (20% B), 30min (25% B), and 50min (25% B; Bian et al., 2011). A flow rate of 1ml/min was used at 25°C, and samples (10μl) were detected at 238nm.

### UPLC-PDA-ESI-MS Analysis of the Main Compositions

The main compositions of the LZF were analyzed using UPLC-MS (Waters H-class QDa). A Waters CORTECS UPLC T3 column (100×2.1mm, 1.6μm) was selected for the separation of the samples. The sample injection volume was 1μl. Through a series of optimization experiments, a mobile phase was eventually adopted for the gradient elution using 25mM ammonium formate with 0.2% formic acid (A) and acetonitrile (B); the specific conditions were: (0–1min) 15–20% B, (1–3min) 20% B, (3–7min) 20–35% B, and (7–8min) 35% B. The detection wavelength was 238nm (scan 210–400nM). The flow rate was 0.25ml/min. The UPLC-MS Quadrupole Dalton (QDa; Waters Corporation) single quadrupole mass spectrometer equipped with electrospray ionization (ESI) was used to record the ESI-MS spectra. The mass spectrometer was operated in the positive and negative ionization mode. The MS analysis method was as follows: The ion spray voltage was set to −0.8kV and +0.8kV in the negative and positive ionization mode, respectively. The turbo-spray temperature was maintained at 600°C. The cone voltage was set at 10 and 50ev to obtain molecular ion peak and secondary fragment, respectively. Both the nebulizer gas (gas 1) and heater gas (gas 2) were set at 50psi, while the curtain gas was kept at 30psi. Nitrogen was used as a nebulizer and auxiliary gas. Samples molecular weight (mw) were collected in both positive and negative modes at the same time (210–650Da).

### Analysis of Microbial

Genomic DNA was extracted *via* the MO BIO PowerSoil DNA Isolation Kit from seven samples (10 and 19days) of the LZF according to manufacturer’s protocols. The ITS2 region and the 16S rDNA V4 region of the sample were amplified by PCR instrument (9700, GeneAmp® ABI, United States; Toju et al., 2012). The purified and diluted genomic DNA as templates and universal primers ITS3 (5′-GATGAAGAACGYAGYRAA-3′), ITS4 (5′-TCCTCCGCTTATTGATATGC-3′) and 515F (5′-GTGYCAGCMGCCGCGGTAA-3′), 806R (5′-GGACTACHVGGGTWTCTAAT-3′) were used for PCR amplification (Caporaso et al., 2011). PCR products were mixed in equal density ratios. Sequencing libraries were generated *via* the TruSeq DNA PCR-Free Sample Prep Kit (Illumina, United States) following manufacturer’s recommendations and index codes were added. Finally, the library was sequenced on an IlluminaHiSeq2500 platform and 250bp paired-end reads were generated. Next-generation sequencing reads were assembled using FLASH (Magoč and Salzberg, 2011). Low quality reads were removed according to the QIIME quality control process (Caporaso et al., 2010, 2011). Sequence analyses were performed *via* Uparse software (Edgar, 2013). A 97% similarity cutoff was used to define operational taxonomic units (OTUs; Gweon et al., 2015). The representative sequences of each OTU were picked and chimeras were removed using Uchime (Edgar et al., 2011). At the same time, the fungal ITS2 region and the bacterial 16S rDNA V4 region were analyzed using the Unite database and the Silva database (Quast et al., 2012), respectively.

### Isolation and Purification of Fermentation Dominant Strains

#### Thermal Stability Test of LZF Fermentation Broth

About 25ml of LZF fermentation broth prepared in section “LZF Preparation and Sampling” were absorbed into 250ml conical flask, and then treated in an autoclave at 121°C for 30min. After cooling, 1ml of each heat-treated sample was extracted into a 10ml volumetric flask, which was volume fixed with methanol and filtered with a 0.22μm filter membrane. Samples were injected and determined according to the HPLC conditions in section “LZF Preparation and Sampling.”

#### Isolation and Purification of the Strain

“Lianzhifan Solution” was directly used as screening medium, solid screening medium was added with appropriate amount of agar. The fermentation broth of 10 and 19days was selected as the sample source of isolates. Around 1ml of fermentation broth was absorbed into 25ml of screening medium at 28 and 37°C at 120rpm, respectively, for enrichment and culture for 3days. Then the enrichment medium was diluted by a 10-fold gradient method according to aseptic operation. About 100μl of diluent was evenly coated on the solid screening medium, and the plate was placed upside down at 28 and 37°C for culture. After the colony grows, single colonies with great differences are selected on each plate and purified according to colony morphology, color, and growth rate, etc., and the purified strains of bacteria and fungi were then stored in test tubes by a streaking method.

#### Screening of Key Strains

The appropriate number of purified strains were picked into the screening medium (LZF) and incubated at 28 and 37°C, 120rpm shaker, respectively, and sampled every 3days to determine the compositional changes by HPLC (*n*=3). The key strains of fermentation were screened according to the magnitude of the conversion rate and conversion rate of gardenia glycosides.

#### Identification of Key Strains

Seven fungal strains obtained from the screening were subjected to genomic DNA extraction according to the instructions of the fungal DNA extraction kit. After the PCR reaction, the amplified products were detected by 1% agarose gel electrophoresis, and then sent to Biotech Bioengineering (Shanghai) for DNA sequencing analysis.

### Evaluation of Virtual Equivalence of Key Strains of Monoculture Fermentation of LZF

#### Evaluation of the Component Equivalence

Seven fungal strains obtained from the screening were activated with Sabouraud’s medium for 2days to obtain spore suspensions. Decoct 1L of LZF according to the recipe, take 250ml into 500ml conical flask, sterilize it with 121°C for 30min, cool it and then inoculate the screened fungal strains, respectively at 10% inoculum, then 28°C, 120rpm for monoculture fermentation. Another 250ml of unsterilized LZF was taken into 500ml beaker and fermented naturally at room temperature. The fermentation was detected by HPLC and set as the end point when the conversion of gardenia glycosides exceeded 95%. Then compare the difference of chemical profile between single bacteria fermentation solution and natural fermentation solution.

#### Evaluation of Pharmacodynamic Equivalence

The perianal abscess rat model was used to evaluate and compare the efficacy of LZF fermented by monoculture of YY46 and YY9 with the natural fermented LZF. Eighteen Wistar male rats of SPF grade, weighing 200±25g, were purchased from Chengdu Dashuo Laboratory Animal Co., Ltd., Ministry of Science and Technology Laboratory Animal Production License No.: SCXK (Chuan) 2015-030. About 2days of acclimatization were kept 8:00–20:00day and night, room temperature 25°C, and free feeding. Referring to the literature method (Zeng et al., 2020), a rat perianal abscess trauma model was constructed. The rats were anesthetized by intraperitoneal injection with 10% chloral hydrate (0.3ml/100g), fixed on the operating table, warming lamp was turned on, disinfected with iodine volt, the hair on both sides of the lower spine of the rats was removed with electric push scissors, local skin preparation was performed, one circular incision of 2cm in diameter was made on the back of the rats, the incision was deep to the muscle layer. After hemostasis, 0.1ml of *Escherichia coli* (OD_600_=1) was applied to the wound surface, and the wound was covered with oil gauze and dressing and fixed with medical tape.

The successful rats were divided into three groups, YY46 group, YY9 group, and LZF group. Six rats were in each group, and the drug was changed once a day, and topical administration was started at 9:00 each day. *Escherichia coli* 0.1ml (OD_600_=1) was added dropwise to the trauma surface 0.5h before the drug change. After rinsing the trauma with saline and iodophor at the time of drug change, 0.5ml of drug solution was added drop by drop, respectively, and the trauma was fixed by applying drug gauze externally. The rats were continuously treated with the drug for 12days. The rats were observed and recorded daily for feeding, body weight, wound color, wound secretion, and the degree of swelling of wound tissue.

The healing rate=(initial wound area−unhealed wound area)/original wound area×100%.

The healing rates of each group were counted and the differences in efficacy were compared.

### Statistical Analysis

The experimental data of the components were analyzed using SPSS (Version 19.0, SPSS Inc., Chicago, IL, United States) software, HemI (Heatmap Illustrator, version 1.0) and Origin Pro 9.0 (OriginLab Corporation, MA, United States), and SIMCA (version 14.1, Umetrics, Umea, Sweden) and SPSS (Version 19.0, SPSS Inc., Chicago, IL, United States) software for compositional and microbial correlation data analysis and Python (version 3.7.1) language for graphing.

## Results

### Analysis of Chemical Profile During the Fermentation of LZF

**Figure 1:**
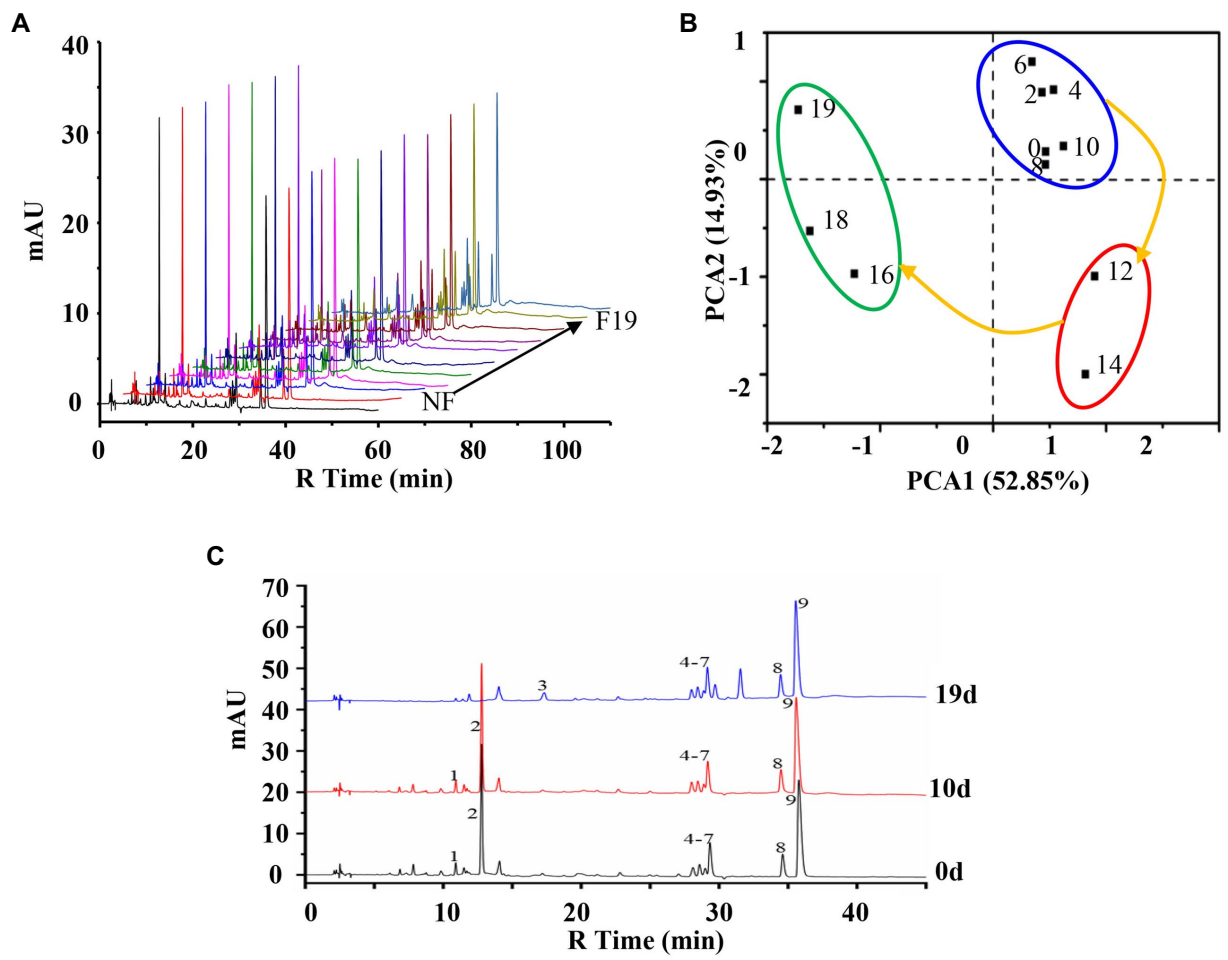
Chemical profile analysis of the fermentation process of “Lianzhifan Solution” (LZF). **(A)** Fingerprints of metabolic profiles at different times of fermentation; **(B)** Principal component analysis (PCA) score plot; **(C)** HPLC chromatograph at 0, 10, and 19days.

A preliminary analysis of the chemical profile of the fermentation process of LZF was carried out by metabolomics. The metabolic profile analysis was performed on the fermentation solution samples taken at different times in the fermentation cycle (0–19days; Figure 1A), and the metabolic profile data were processed by principal component analysis (PCA) score plot (Figure 1B), PCA1 and PCA2 were 52.85 and 14.93%, respectively, which can fully explain the differences among samples. It can be seen from the distances between different sample points that the fermentation process of LZF can be divided into three stages: early stage of fermentation (ESF; 0–10days), medium stage of fermentation (MSF; 12–14days), and last stage of fermentation (LSF; 16–19days).

According to the method of peak area normalization, the chromatographic peaks of chemical components accounting for more than 2% in the fermentation broth at 0, 10 and, 19days were counted. The results showed that there were nine main chromatographic peaks. The peak area of compounds 1 and 2 did not change significantly at the early stage of fermentation, but began to decline from the middle stage of fermentation, and gradually decreased to 0 at the later stage. Compound 3 is a newly generated component after fermentation. During the whole fermentation process, the peak area of compounds 4–9 did not change significantly, among which compound 9 had the largest peak area (Figure 1C).

### Confirmation and Changes of the Major Chemical Components During the Fermentation of LZF

**Figure 2:**
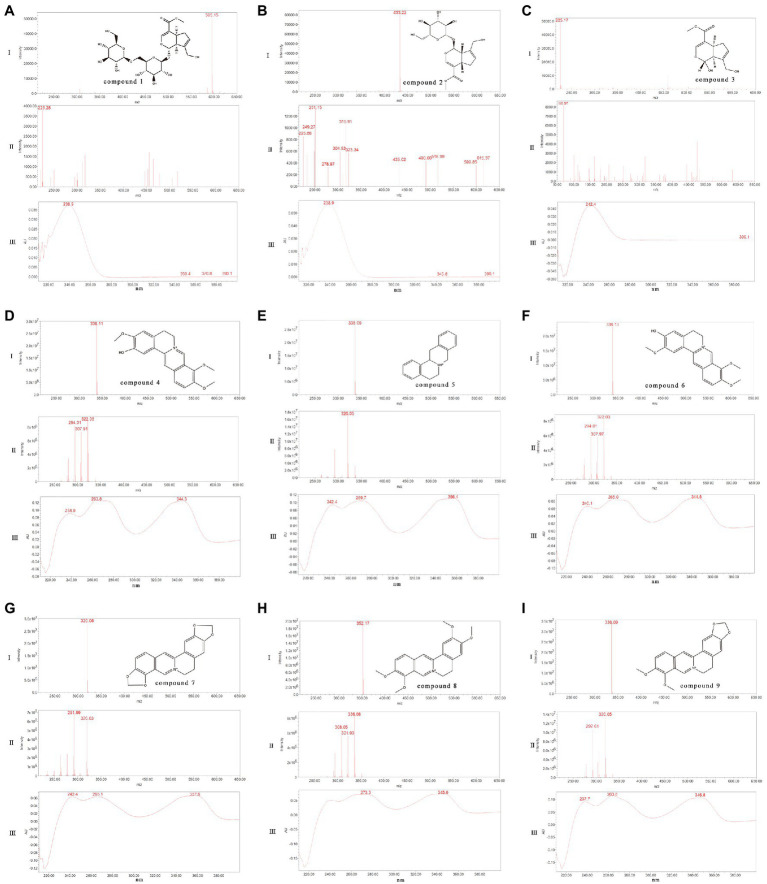
Identification of the main chemical components in the fermentation process of LZF [I: UPLC-ESI-MS Spectrum (10ev); II: UPLC-ESI-MS Spectrum (50ev); III: UPLC-PDA Spectrum (210–400nm)]. **(A)** Compound 1: Genipin-1-*β*-D-gentiobioside; **(B)** Compound 2: Geniposide; **(C)** Compound 3: Genipin; **(D)** Compound 4: Columbamine; **(E)** Compound 5: Epiberberine; **(F)** Compound 6: Jatrorrhizine; **(G)** Compound 7: Coptisine; **(H)** Compound 8: Palmatine; and **(I)** Compound 9: Berberine.

The main formulations of LZF are Coptis and Gardenia, where the main active ingredient of Coptis is benzyl isoquinoline alkaloid; the main active ingredient of Gardenia is iridoid glycosides. The main nine compounds in the solution were further confirmed by UPLC-PDA-ESI-MS method. We compared the quasi-molecular ion peaks, fragment ion peaks, and spectral features of the compounds with the reported UV spectra and mass spectral data. Nine compounds were presumed to be genipin-1-*β*-D-gentiobioside, geniposide, genipin, columbamine, epiberberine, jatrorrhizine, coptisine, palmatine, and berberine. The corresponding controls were then analyzed in the same way, and then the nine compounds were further confirmed by comparing the mass spectrometry data and retention times (Rtime), and the results are shown in Figure 2.

Compound 1: Genipin-1-*β*-D-gentiobioside. Molecular formula is C_23_H_34_O_15_. Relative molecular mass is 550. In negative ion mode, ion fragmentation was *m*/*z* 595 [M+CHO_2_]^−^ (10ev), *m*/*z* 549 [M-H]^−^(10ev), and the deglycoside parent nucleus (genipin) ion fragmentation *m*/*z* 225 (50ev). The maximum absorption wavelength was 238.9nm, which was consistent with control quality spectrum data.
Compound 2: Geniposide. Molecular formula is C_17_H_24_O_10_. Relative molecular mass is 478. In negative ion mode, ion fragmentation was *m*/*z* 477 [M+CHO_2_]^−^(10ev) and the deglycoside parent nucleus (genipin) ion fragmentation *m*/*z* 225 (50ev). The maximum wavelength was 238.9nm, which was consistent with the control quality spectrum data.
Compound 3: Genipin. Molecular formula is C_11_H_14_O_5_. Relative molecular mass is 226. In negative ion mode, ion fragmentation was *m*/*z* 225 [M-H]^−^(10ev), *m*/*z* 69 (50ev). The maximum absorption wavelength was 244.8nm, which was consistent with the control quality spectrum data.
Compound 4: Columbamine. Molecular formula C_20_H_20_NO_4_, relative molecular mass 338. In positive ion mode, ion fragmentation was *m*/*z* 338 [M]^+^ (10ev), *m*/*z* 323 [M-CH_3_]^+^ (50ev); *m*/*z* 308 [M-CH_3_-CH_3_]^+^ (50ev), *m*/*z* 294 [M-CH_3_-H-CO]^+^ (50ev), and *m*/*z* 280 [M-CH_3_-CH_3_-CO]^+^ (50ev). The maximum absorption wavelength 263.8nm (100%), 344.5nm (99%), which were consistent with the control quality spectral data.
Compound 5: Epiberberine. Molecular formula is C_20_H_18_NO_4_. Relative molecular mass is 336. In positive ion mode, ion fragmentation was *m*/*z* 336 [M]^+^ (10ev), *m*/*z* 320 [M-CH_3_]^+^ (50ev), *m*/*z* 292 [M-CH_3_-H-CO]^+^ (50ev). The maximum absorption wavelength 269.7nm (100%), 356.4nm (99%), and 265.0nm (99%), which were consistent with the data of control quality spectrum.
Compound 6: Jatrorrhizine. Molecular formula is C_20_H_20_NO_4_. Relative molecular mass is 338. In positive ion mode, ion fragmentation was *m*/*z* 338 [M]^+^ (10ev), *m*/*z* 323 [M-CH_3_]^+^ (50ev), *m*/*z* 294 [M-CH_3_-H-CO]^+^ (50ev), and *m*/*z* 280 [M-CH_3_-CH_3_-CO]^+^ (50ev). The maximum absorption wavelength is 344.5nm (100%) and 265.0nm (99%), which were consistent with the data of control quality spectrum.
Compound 7: Coptisine. Molecular formula is C_19_H_14_NO_4_, Relative molecular mass is 320. In positive ion mode, ion fragmentation was *m*/*z* 320 [M]^+^ (10ev), *m*/*z* 292 [M-CO]^+^ (50ev); *m*/*z* 277 [M-CH_2_O-CH]^+^ (50ev) *m*/*z*, *m*/*z* 262 [M-CH_2_O-CO]^+^ (50ev), and *m*/*z* 249 [M-CH_2_O-CH-CO]^+^ (50ev). The maximum absorption wavelengths were 357.6nm (100%) and 266.1nm (99%), which were consistent with the control quality spectral data.
Compound 8: Palmatine. Molecular formula is C_21_H_22_NO_4_, Relative molecular mass is 352. in positive ion mode, ion fragmentation was *m*/*z* 352 [M]^+^ (10ev), *m*/*z* 337 [M-CH_3_]^+^ (50ev); *m*/*z* 322 [M-CH_3_-CH_3_]^+^ (50ev), *m*/*z* 308 [M-CH_3_-H-CO]^+^ (50ev), and *m*/*z* 294 [M-CH_3_-CH_3_-CO]^+^ (50ev). The maximum absorption wavelength were 344.5nm (100%) and 273.3nm (100%), which were consistent with the control quality spectral data.
Compound 9: Berberine. Molecular formula is C_20_H_18_NO_4_. Relative molecular mass is 336. In positive ion mode, ion fragmentation was *m*/*z* 336 [M]^+^ (10ev), *m*/*z* 320 [M-CH_3_-H]^+^ (50ev); *m*/*z* 306 [M-CH_3_-CH_3_]^+^ (50ev), *m*/*z* 292 [M-CH_3_-H-CO]^+^ (50ev), and *m*/*z* 278 [M-CH_3_-CH_3_-CO]^+^ (50ev). The maximum absorption wavelength were 263.8nm (100%) and 346.8nm (100%), which were consistent with the control quality spectrum data.

**Figure 3:**
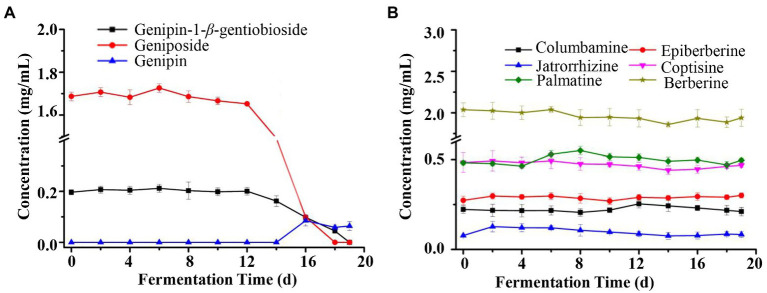
Chemical composition dynamic changes of LZF after fermentation. **(A)** Content of iridoids (Genipin-1-*β*-D-gentiobioside, Geniposide, and Genipin) during fermentation of LZF; **(B)** Content of alkaloids during fermentation of LZF.

Therefore, further analysis of the characteristics of the changes of these nine compounds throughout the fermentation process showed that there was a significant biotransformation of genipin-1-*β*-D-gentiobioside and geniposide, and their content changes were not significant in the early stage of fermentation, and started to decrease from the 12days of fermentation, and decreased to near trace levels in the final stage of fermentation (Figure 3A). Genipin was a newly formed composition, and started to increase from the 14days of fermentation, and stabilized in the final stage of fermentation. This correlated with the decreasing trend of the contents of genipin-1-*β*-D-gentiobioside and geniposide, so genipin-1-*β*-D-gentiobioside and geniposide were converted into genipin by microbial conversion. At the same time, the contents of six alkaloids, including columbamine, epiberberine, jatrorrhizine, coptisine, palmatine, and berberine, did not change significantly during the whole fermentation process, with almost no obvious biotransformation (Figure 3B). Therefore, iridoid glycosides were the main substances of biotransformation in the fermentation process, and alkaloids were relatively stable at the initial level.

### Correlation Between Dominant Microbiome and Compound Transformation in LZF Fermentation

**Figure 4:**
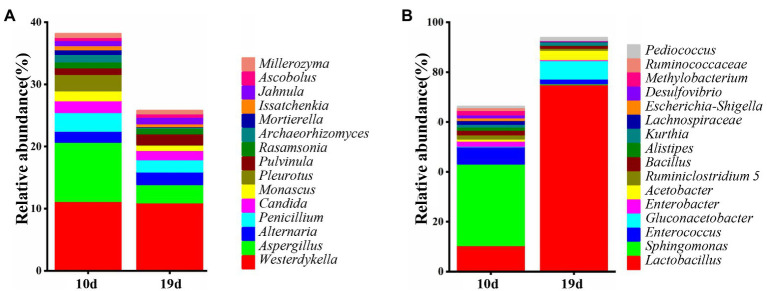
Distribution of the fungi **(A)** and bacteria **(B)** during fermentation process.

Microorganisms can transform the main components in the fermentation, so we analyzed the dominant bacteria and fungi in the fermentation process using high-throughput sequencing technology. The community composition and abundance of species in the LZF fermentation broth at 10 and 19days were analyzed at the genus level, and the results are shown in Figure 4. As can be seen, there were 15 dominant genera of fungi (relative abundance>0.5%), which were *Westerdykella* (11.08%), *Aspergillus* (6.22%), *Alternaria* (1.91%), *Penicillium* (2.50%), *Candida* (1.70%), and *Monascus* (1.19%), *Pleurotus* (1.38%), *Pulvinula* (1.41%), *Rasamsonia* (0.96%), *Archaeorhizomyces* (0.65%), *Mortierella* (0.42%), *Issatchenkia* (0.57%), *Jahnula* (0.94%), *Ascobolus* (0.53%), and *Millerozyma* (0.52%). There were 16 dominant bacterial genera (relative abundance>1%) and they were *Lactobacillus* (42.80%), *Sphingomonas* (16.53%), *Enterococcus* (4.40%), *Gluconacetobacter* (3.80%), *Enterobacter* (1.23%), *Acetobacter* (2.29%), *Ruminiclostridium* 5 (1.28%), *Bacillus* (1.50%), *Alistipes* (0.67%), *Kurthia* (1.20%), *Lachnospiraceae* NK4A136 group (0.87%), *Escherichia-Shigella* (0.61%), *Desulfovibrio* (0.67%), *Methylobacterium* (0.92%), *Ruminococcaceae* NK4A214 group (0.59%), and *Pediococcus* (0.71%).

**Figure 5:**
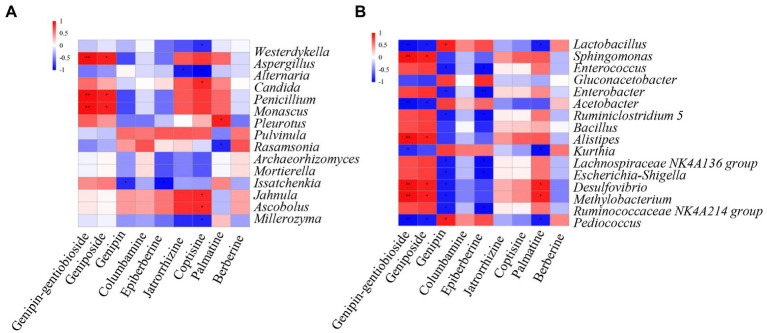
Spearman correlation heatmap between dominant **(A)** fungal and **(B)** bacterial genera and chemical compound during LZF fermentation. The *X* and *Y* axes are chemical compound and abundant fungal and bacterial genera, respectively. The correlation coefficient (*R*) appears in different colors. The left side of the legend is the color range of different *R* values. The red color represents positive correlation and the blue color represents negative correlation. Significant values are shown as: ^*^p>0.01; ^**^p>0.001.

To analyze the correlation between the dominant genera of LZF fermentation and compound biotransformation, we calculated Spearman correlation coefficients of 15 dominant fungal genera and 16 dominant bacterial genera with compounds and plotted Spearman correlation heat map with R software (Figure 5). As can be seen, the fungal genera *Aspergillus*, *Penicillium*, and *Monascus* were highly significantly positively correlated with genipin-1-*β*-D-gentiobioside (*p* <0.01) and with geniposide (*p* <0.05). Coptisine was significantly positively correlated with *Jahnula*, *Ascobolus*, and *Candida* significantly positively correlated (*p* <0.05) and with *Westerdykella*, *Millerozyma*, and *Alternaria* significantly negatively correlated (*p* <0.05). Bacteria were more correlated with iridoids glycosides components in LZF, *Sphingomonas*, *Alistipes*, and *Desulfovibrio*, *Methylobacterium* were highly significantly positively correlated with genipin-1-*β*-D-gentiobioside (*p* <0.01) and with geniposide (*p* <0.05); *Lactobacillus*, *Acetobacter*, and *Pediococcus* were highly significantly negatively correlated with genipin-1-*β*-D-gentiobioside (*p* <0.01) and with geniposide (*p* <0.05). Genipin was significantly positively correlated with *Lactobacillus* and *Pediococcus* significantly positively correlated (*p* <0.05) and significantly negatively correlated (*p* <0.05) with dominant bacteria such as *Enterococcus*, *Enterobacter*, and *Ruminiclostridium*.

### Isolation and Identification of Dominant Strains

The thermal stability of the LZF pre-fermentation decoction was evaluated according to the chromatographic conditions in section “LZF Preparation and Sampling,” and no chemical profile changes occurred before and after sterilization. We used this decoction as selection media to screen the transformation ability of isolated and purified bacterial and fungal strains, and artificially inoculated these strains for monoculture fermentation for 15days after sampling to detect the changes of nine main components. The results of the chemical composition in the fermentation broth of bacterial strains were unchanged and could not transform the iridoids, but the fermentation broth of fungal strains mostly showed changes in composition. Seven of the fungal strains (named YY-5, YY-9, YY-19, YY-23, YY-34, YY-46, and YY-51) were selected for identification by combining growth rate and conversion ability, and the results were identified as *Aspergillus japonicus* YY-5, *Aspergillus niger* YY-9, *Cladosporium oxysporum* YY-19, *Cladosporium tenuissimum* YY-23, *Cladosporium cladosporioides* YY-34, *Penicillium expansum* YY-46, and *Penicillium qlabrum* YY-51.

**Figure 6:**
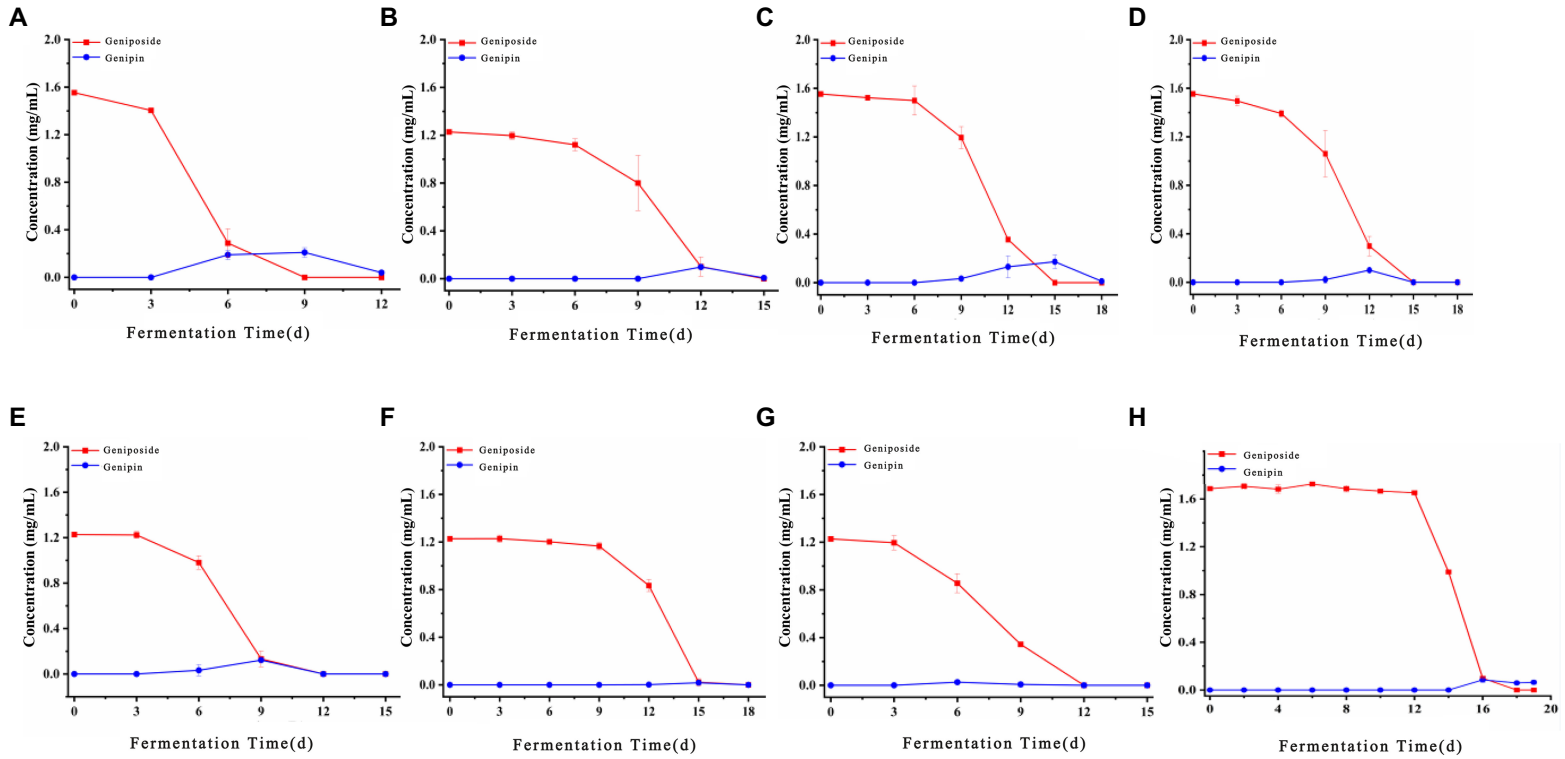
Content of iridoids during fermentation of LZF by monoculture fermentation and natural fermentation. **(A)** *Penicillium expansum* YY-46; **(B)** *Penicillium qlabrum* YY-51; **(C)** *Aspergillus niger* YY-9; **(D)** *Aspergillus japonicus* YY-5; **(E)** *Cladosporium oxysporum* YY-19; **(F)** *Cladosporium tenuissimum* YY-23; **(G)** *Cladosporium cladosporioides* YY-34; and **(H)** natural fermentation.

**Figure 7:**
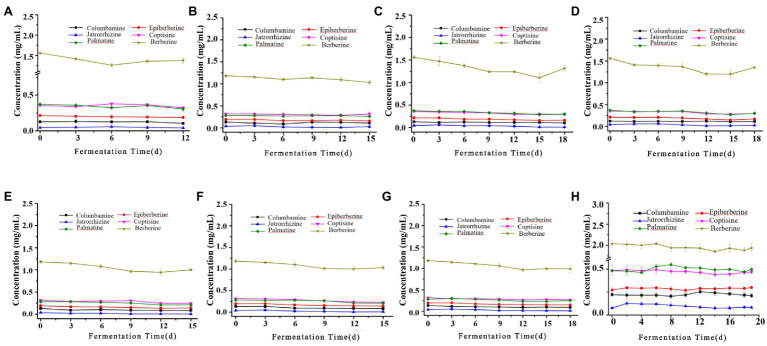
Content of alkaloids during fermentation of LZF by monoculture fermentation and natural fermentation. **(A)** *Penicillium expansum* YY-46; **(B)** *P. qlabrum* YY-51; **(C)** *A. niger* YY-9; **(D)** *A. japonicus* YY-5; **(E)** *C. oxysporum* YY-19; **(F)** *C. tenuissimum* YY-23; **(G)** *C. cladosporioides* YY-34; and **(H)** natural fermentation.

The biotransformation ability of seven fungal strains was further compared, as shown in Figures 6, 7. The dynamics of the components of the LZF fermented by *P. expansum* YY-46, *Penicillium qlabrum* YY-51, *A. niger* YY-9, and *Aspergillus japonicus* YY-5 were consistent with those of the natural fermentation process of the solution, while the dynamics of the components of the LZF fermented by *C. oxysporum* YY-19, *C. tenuissimum* YY-23, and *C. cladosporioides* YY-34 fermentation showed some differences in compositional changes from those during natural fermentation. In terms of the biotransformation ability of gardenia glycosides, all seven strains were able to achieve 100% conversion, but the conversion rate varied among strains. *Penicillium expansum* YY-46 had the strongest transformation ability and the fastest conversion rate, and the conversion rate had reached 100% at 9days. *Cladosporium tenuissimum* YY-23 had the slowest conversion rate, with complete conversion at 18days. Therefore, in order of transformation rate, the transformation capacity of seven strains was *P. expansum* YY-46>*A. niger* YY-9>*A. japonicus* YY-5>*P. qlabrum* YY-51>*C. oxysporum* YY-19>*C. cladosporioides* YY34>*C. tenuissimum* YY-23. Extended *P. japonicum* YY-46 and *A. niger* YY-9 were the two fermentative strains with high transformation capacity. The results were also consistent with the correlation analysis and the observation of natural fermentation, so *P. expansum* YY-46 and *A. niger* YY-9 were identified as the key strains for the fermentation of LZF.

### Evaluation of Virtual Equivalence of Key Strains of Monoculture Fermentation of LZF

#### Evaluation of the Composition Equivalence

**Figure 8:**
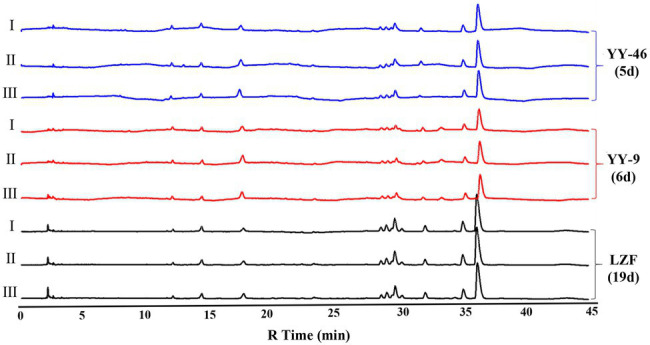
HPLC chromatogram of chemical component equivalence evaluation (I, II, and III are parallel samples).

About 3L LZF was decocted according to the formula, and 250ml was taken into a 500ml conical flask. After sterilization at 121°C for 30min, strains YY-46 and YY-9 were inoculated according to the aseptic operation after cooling, and then monoculture fermentation was conducted at 28°C and 120rpm. Another 250ml of unsterilized LZF was taken into a 500ml beaker and fermented naturally at room temperature. Samples were taken daily and detected by HPLC. When the conversion of gardenoside exceeded 95%, it was the end point of fermentation. There were three samples in parallel in each group. Our results indicated that the fermentation ending time of YY-46 and YY-9 strains was 5 and 6days respectively, while the fermentation ending time of natural process was 19days (Figure 8). Furthermore, the metabolic profiles of LZF prepared by two kinds of single bacteria were matched with those of LZF prepared by natural method, and the similarity evaluation was carried out. The similarity between these two metabolic profiles ranged from 0.963 to 1.000. The results indicated that the chemical compositions of the solution prepared by the monoculture and the natural preparation were essentially homogeneous. However, when the inoculation amount and fermentation conditions were optimized, the conversion of iridoid glycosides by monoculture fermentation was greatly improved. The fermentation cycle of YY-46 was 5days, which was nearly four times shorter than natural fermentation cycle, and the fermentation cycle of YY-9 was 6days, which was also nearly three times shorter than natural fermentation cycle. Therefore, monoculture fermentation can shorten the fermentation cycle and improve the fermentation efficiency under the condition of consistent quality, and the fermentation process for mass production can be further optimized in the later stage. The results are shown in Table 1.

**Table 1:** Similarity analysis of three kinds of LZF.

	LZF-1	LZF-2	LZF-3	YY-9-1	YY-9-2	YY-9-3	YY-64-1	YY-64-2	YY-64-3
LZF-1	1.000	0.999	0.999	0.983	0.968	0.979	0.982	0.99	0.993
LZF-2	0.999	1.000	1.000	0.98	0.964	0.976	0.980	0.988	0.994
LZF-3	0.999	1.000	1.000	0.978	0.963	0.976	0.979	0.987	0.993
YY-9-1	0.983	0.980	0.978	1.000	0.996	0.996	0.993	0.993	0.989
YY-9-2	0.968	0.964	0.963	0.996	1.000	0.995	0.990	0.986	0.979
YY-9-3	0.979	0.976	0.976	0.996	0.995	1.000	0.987	0.989	0.988
YY-64-1	0.982	0.980	0.979	0.993	0.990	0.987	1.000	0.996	0.992
YY-64-2	0.990	0.988	0.987	0.993	0.986	0.989	0.996	1.000	0.997
YY-64-3	0.993	0.994	0.993	0.989	0.979	0.988	0.992	0.997	1.000

#### Evaluation of Pharmacodynamics

**Figure 9:**
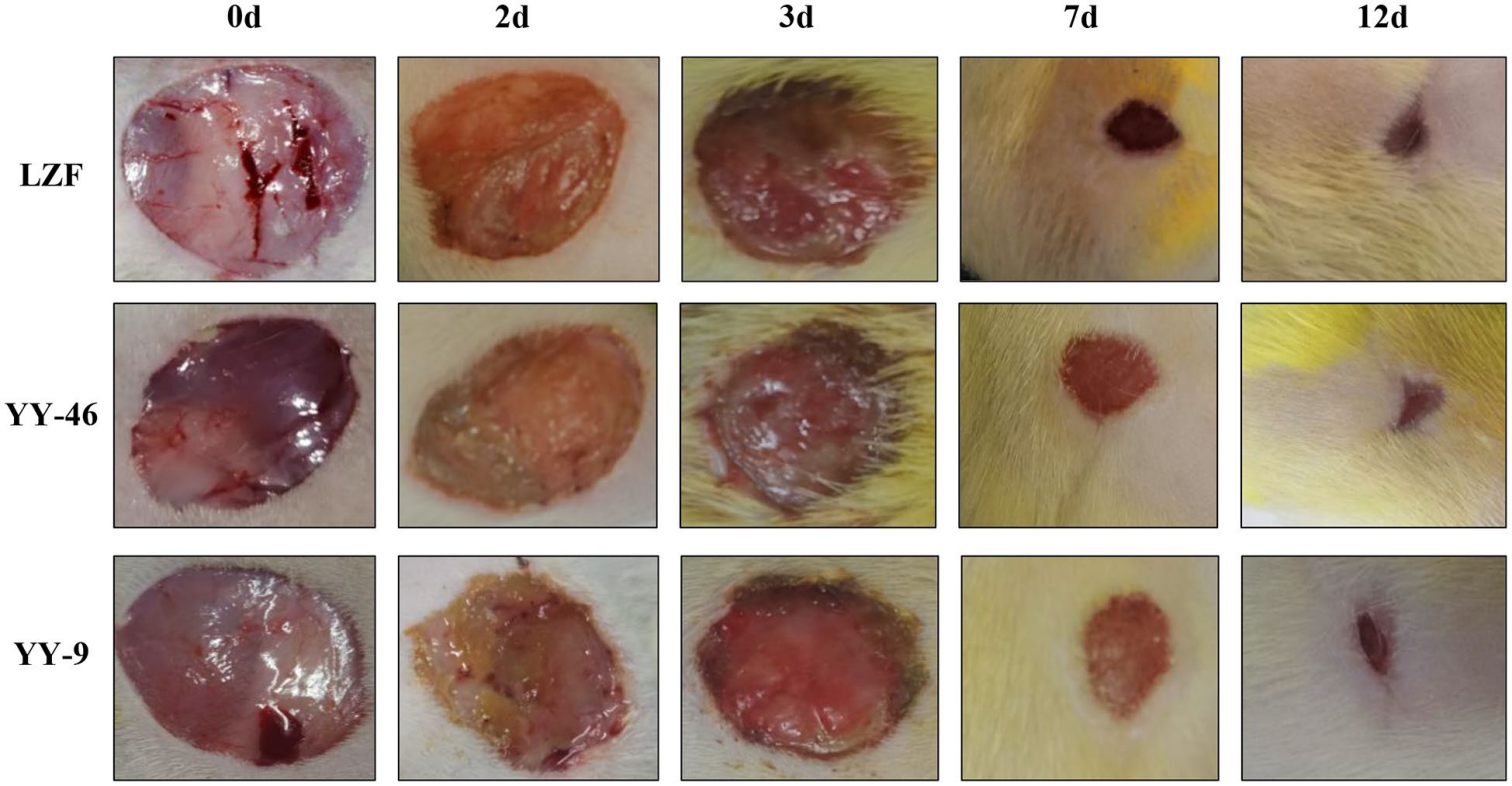
Equivalence evaluation of pharmacodynamics in rats.

A rat model of perianal abscess was used to evaluate and compare the efficacy of YY46, YY9, and LZF groups. Morphological observations showed that the degree of wound oozing, skin swelling, and redness around the wound edges of rats in the LZF and YY46 groups were basically the same during the treatment process, and the YY9 group showed a slightly weaker therapeutic effect than the other two groups, but there was no significant difference. At 7days, the exudate almost disappeared, the granulation tissue started to crust and a small amount of blood leaked when crust was touched by cotton swabs in all treatment groups. At 12days, there was no significant exudate and epithelial coverage in the treated group; the wound was smaller in the LZF and YY46 groups than in the YY9 group, as shown in Figure 9. After 12days of treatment, the rats in the YY46 and LZF groups recovered better, and the wound healing rate had reached 94% by 12days. Although, the wound healing rate of the YY9 group was lower than that of the other two groups throughout the observation period, it also reached 92% healing rate at 12days (Table 2). Therefore, both of the two new preparations of monoculture fermentation with LZF could promote the healing of perianal abscess wounds in rats, and had virtual pharmacodynamic equivalence with the traditional preparation.

**Table 2:** Effect of three kinds of LZF on the wound healing rate of rats (*n*=6, *X*±*s*)

Groups	Wound healing rate (M±*SD*%)		
	Day 3	Day 7	Day 12
LZF	23.67±6.07	85.69±1.51	94.28±1.08
YY46	37.54±3.86	85.44±1.88	94.03±2.79
YY9	29.22±5.54	79.37±6.26	92.49±5.27

## Discussion

Natural fermentation is one of the traditional methods of processing herbal preparations and has a long history in China, while modern pharmaceutical industries commonly use monoculture fermentation for mass production of small molecule drugs (Wang et al., 2019; Zhang et al., 2021). The fermentation process is often accompanied by the change of chemical profile, but some chemical composition remains stable (Gao et al., 2020; Cai et al., 2021). We found a similar phenomenon in LZF fermentation. A variety of iridoids glycosides (compound 1 and 2) were converted to genipin (compound 3) by the action of various glycosidases secreted by microbiome, while alkaloids (compound 4–9) with antibacterial effects maintained compositional stability before and after fermentation. These results suggested the coexistence of instable and stable chemical composition in the fermentation system, and the biotransformation of instable composition should be the main event of the fermentation. Therefore, we speculate that iridoid deglycosylation is a key event in LZF fermentation.

During the fermentation process, the structural evolution of microbial communities was accompanied by significant changes in the chemical profile (An et al., 2020; Meng et al., 2021). High-throughput sequencing technology is developed to dissect microecological structures in several fields, such as food (Wu et al., 2019), healthcare (Fernanda et al., 2018), and environment (Guo et al., 2019), and it is also a frontier technology for screening microorganisms in complex microbial fermentation system. Recently, high-throughput sequencing and metabolomics have been used to predict the key strains of fermentation by correlation analysis, but researchers also believe that the predicted key strains would include culture-dependent and culture-independent strains (Xu et al., 2020, 2021). Our results showed that bacteria had a greater correlation with chemical composition in LZF fermentation than fungi did. However, when we focused on the key events of LZF fermentation and used classical screening methods, we found that fungal strains had a greater contribution to the biotransformation events than bacteria. Thus, the true key microbial strains remain to be determined and confirmed in both predictable and real fermentation systems. Therefore, only in the real fermentation system, we can obtain the identified strains by focusing on the main events of fermentation to screen the key strains.

Deglycosylation that occurs during the fermentation of LZF produces genipin, which has anti-inflammatory and antioxidant biological activities and play a role in promoting wound healing through anti-inflammatory pathways, while the hydrophilicity of iridoids glycosides is higher than the lipophilicity, thus making them difficult to exert their medicinal effects transdermally (Shanmugam et al., 2018). The deglycosylation reduce genipin’s hydrophilicity and enhance its lipophilicity, so as to achieve transdermal properties to exert the pharmacodynamics effects. After obtaining two highly deglycosylated biotransformed strains, we performed monoculture fermentation to evaluate the equivalence of chemical component between the monoculture fermentation preparation and the traditional fermentation preparation. The results suggested that the monoculture fermentation preparations and the traditional fermentation had a chemical component equivalence. The new preparations also maintain the characteristics of alkaloid components stable, while making iridoids glycosides deglycosylation unstable. Therefore, we can speculate that these two strains may be the key strains in the LZF fermentation. However, the equivalence of the main chemical components of the natural medicine does not directly infer that the new preparations are equivalent to the traditional preparation in terms of biological activity. It is necessary to evaluate and validate the bioactive equivalence in the real world. Therefore, in our strategy of screening key functional strains, we added the important aspect of bioactivity equivalence evaluation as a post-verification operation step for the purpose of efficacy. The evaluation results were in accordance with expectations, and the new and traditional preparations were virtual equivalent in the treatment of perianal abscess. Therefore, by analyzing microfauna communities-chemical component in fermentation systems, and then evaluating the bioactivity of new preparations for virtual equivalence by purified strains, a “Microfauna communities-Chemical component-Pharmacodynamic” axis is formed for screening key functional strains in complex fermentation systems, which can realize a new strategy for complete combination of high-throughput screening theory and real-world reassessment.

## Conclusion

In this study, the chemical component and bioequivalence evaluation proved that the monoculture fermentation was more effective than the natural fermentation, suggesting that this method could be used as an upgrade measure for the production process. We sought to determine whether it is possible to define a predictive strategy based on “Microfauna communities-Chemical component-Pharmacodynamic” axis that can be used to select key strains who can reduce the time of process and preserve the initial metabolites. This strategy not only provides new ideas for TCM fermentation preparation technology, but also provides an important scientific basis for improving the production process and formulating GMP work. More importantly, it is also applicable to the whole fermentation drugs industry.

### Accession Number of Sequences

The sequence information from pyrosequencing has been uploaded to the European Nucleotide Archive database under the accession number PRJEB35333.

## Data Availability Statement

The datasets presented in this study can be found in online repositories. The names of the repository/repositories and accession number(s) can be found at: https://www.ebi.ac.uk/ena, PRJEB35333.

## Ethics Statement

The animal study was reviewed and approved by the Ethics Committee of Sichuan Normal University.

## Author Contributions

JX, YY, and XZ designed the study. YY conducted the experiment, analyzed the data, and with JX drafted the manuscript. XYa and XYu provided experimental conditions. TP prepared the experimental materials. TP and QP helped to revise the manuscript. All authors contributed to the article and approved the submitted version.

## Conflict of Interest

TP was employed by company Keystonecare Technology (Chengdu) Co.

The remaining authors declare that the research was conducted in the absence of any commercial or financial relationships that could be construed as a potential conflict of interest.

## Publisher’s Note

All claims expressed in this article are solely those of the authors and do not necessarily represent those of their affiliated organizations, or those of the publisher, the editors and the reviewers. Any product that may be evaluated in this article, or claim that may be made by its manufacturer, is not guaranteed or endorsed by the publisher.
